# Inhibition of epigenetic regulator UHRF1 attenuates renal fibrosis and retains transcription factor Krüppel-like factor 15 expression

**DOI:** 10.1038/s41420-025-02549-y

**Published:** 2025-06-09

**Authors:** Yulu Gu, Shiqi Lv, Xinhui Huang, Jialin Wang, Yulin Wang, Han Zhang, Ziyan Shen, Jing Chen, Cheng Zhu, Di Zhang, Xiaoqiang Ding, Xiaoyan Zhang

**Affiliations:** 1 Department of Nephrology, Zhongshan Hospital, Fudan University, Shanghai, China; 2 Division of Nephrology, The Second People’s Hospital of Changzhou, The Third Affiliated Hospital of Nanjing Medical University, Changzhou Medical Center, Nanjing Medical University, Jiangsu, China; 3 Department of Nephrology, Huadong Hospital, Fudan University, Shanghai, China; 4 Shanghai Medical Center of Kidney Disease, Shanghai, China; 5 Shanghai Key Laboratory of Kidney and Blood Purification, Shanghai, China; 6 Shanghai Institute of Kidney and Dialysis, Shanghai, China

**Keywords:** Renal fibrosis, DNA methylation

## Abstract

Aberrant DNA methylation modification is well-known to be involved in renal fibrogenesis. As a critical cooperator in DNA methyltransferase 1 (DNMT1)-mediated maintenance of DNA methylation, the role of ubiquitin-like containing PHD and RING finger domains 1 (UHRF1) in renal fibrosis remains unknown. Here, upregulation of UHRF1 is observed in activated renal fibroblasts. Fibroblasts-specific depletion of UHRF1 reduces fibrotic lesions in both unilateral ureter obstruction- and unilateral renal ischemia-reperfusion injury-induced murine models of kidney fibrosis. Through Reduced Representation Bisulfite Sequencing, Krüppel-like factor 15 (KLF15) is screened and further verified as the target methylated gene of UHRF1 and responsible for fibroblasts activation. Moreover, UHRF1 induces KLF15 methylation through interacting with DNMT1. Genetic depletion of UHRF1 or pharmacological inhibition of such interaction decreases KLF15 methylation levels and restores its expression, resulting in reduced renal fibroblasts activation and kidney fibrosis. Collectively, these results suggest that UHRF1 may be a promising target for mitigating renal fibrosis.

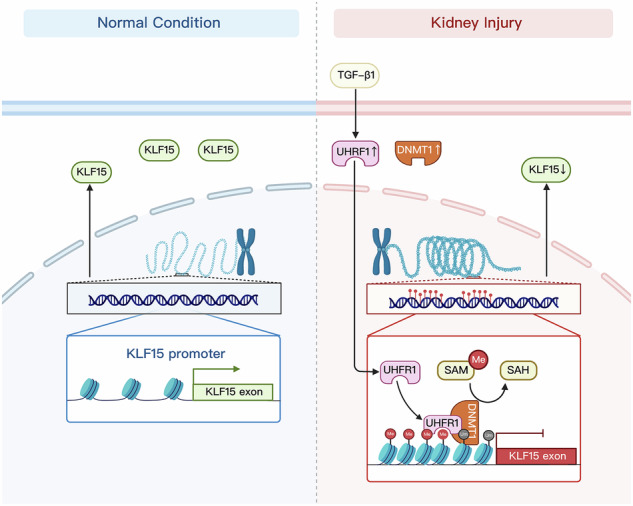

## Introduction

Renal fibrosis is the final common pathway leading to end-stage renal disease (ESRD), irrespective of its initial causes. Fibroblasts are the major source for myofibroblasts in fibrogenesis. And fibroblasts activation is considered as the hallmark of renal tubulointerstitial fibrosis [1, 2]. Epigenetic modifications have been reported to regulate fibroblasts activation, especially DNA methylation [3]. DNA methylation is defined as addition of a methylgroup to the 5-position of cytosine catalyzed by DNA methyltransferase (DNMT). DNA methylation in promotor regions contributes to transcriptional inactivation. Recent studies have revealed that promotor hypermethylation of multiple genes including *RASAL1*, *PPARα*, *PPARγ and SFRP3*, is strongly associated with fibroblasts activation [3–6]. Based on these, several demethylating agents in slowing the progression of renal fibrosis exert their therapeutic potential in various animal models [3, 7, 8]. However, it remains challenging to put them into clinical use for their side effects. Also, further effort is required to understand the molecular mechanism about how target genes are methylated in progression of renal fibrosis.

Gene methylation is established in early development by DNMT3A and DNMT3B, called as de novo methylation. Once established, DNA methylation patterns are maintained by DNMT1 during DNA replication, called as maintenance methylation [9, 10]. DNA maintenance methylation plays a crucial role in renal fibrosis. Inhibition of DNMT1 expression or activity contributes to improvement of renal fibrosis [3, 8]. Such drugs including 5-Azacytidine have considerable cytotoxicity due to DNA-incorporation [11, 12].

Ubiquitin-like containing PHD and RING finger domains 1 (UHRF1), as a cooperator of DNMT1, is essential in maintenance methylation [13]. During DNA replication, UHRF1 specifically recognizes hemimethylated DNA, and recruits DNMT1 to targeted sites via its SRA domain, eventually making sure the methylation of newly-synthesized strand [14, 15]. Previous research has reported that depletion of UHRF1 causes loss of global DNA methylation or decreased level of target genes methylation [13, 16–18]. But little is known about the role of UHRF1 in renal fibrogenesis.

In this study, we found UHRF1 was upregulated in fibrotic renal fibroblasts. To better understand the role of UHRF1 in progression of renal fibrosis, we performed Reduced Representation Bisulfite Sequencing (RRBS) in nonfibrotic fibroblasts and fibrotic fibroblasts. Among the differentially methylated regions (DMRs), we screened KLF15, which was reported to ameliorate renal fibrosis and fibroblast activation. Our resulting data further showed that UHRF1 contributed to progression of renal fibrosis via methylating KLF15 promotors depending on its interaction with DNMT1, implying that inhibition of UHRF1 or its interaction with DNMT1 could be a novel and effective target for the treatment of renal fibrosis.

## Results

### UHRF1 expression is increased in fibrotic renal fibroblasts

**Fig. 1:**
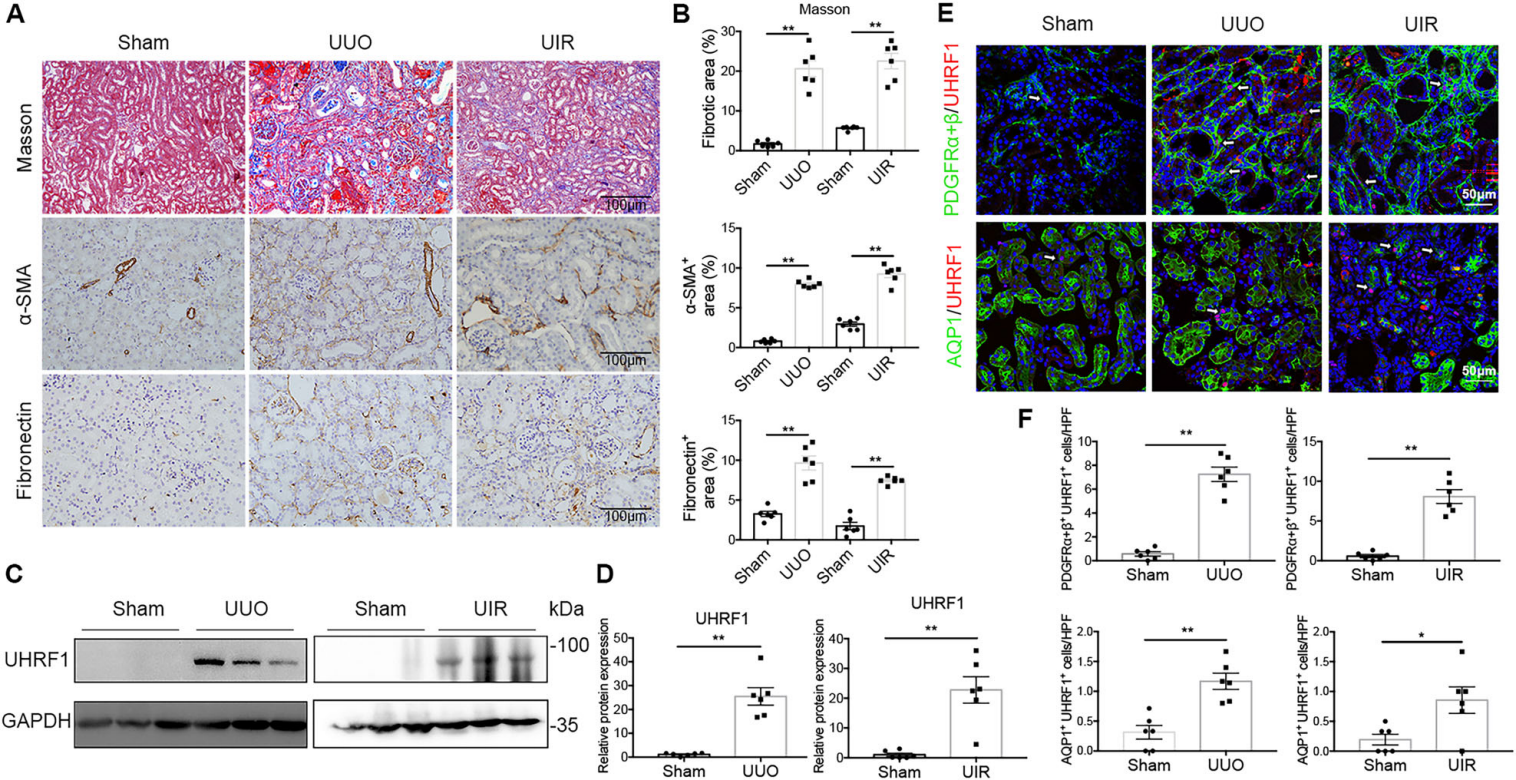
Upregulation of UHRF1 in fibrotic kidney fibroblasts. **A** Masson staining (×200) and immunochemistry staining (×200) for α-SMA and Fibronectin on days 7 in UUO mice and on days 14 in UIR mice. Scale bar: 100 μm. **B** The bar graph summarizes the relative fibrotic area or α-SMA-positive and Fibronectin positive areas in each group. **C**, **D** Relative renal KLF15 protein expression in UUO mice or in UIR mice, analyzed by western blots. **E**, **F** Immunofluorescence double-labeling with antibodies to UHRF1 (Red) and PDFGRα + β (green) or AQP1 (green) (×200). The white arrow represents UHRF1^+^ fibroblasts (top panel) and UHRF1^+^ proximal tubular epithelial cells (bottom panel). Scale bar: 50 μm. *n* = 6 mice per group. Data were shown as mean ± SEM. Data were analyzed using unpaired Student’s *t* test. *P < 0.05, **P < 0.01.

UHRF1 plays an important role in DNA CpG methylation, heterochromatin function and gene expression. To ascertain the possible involvement of UHRF1 in renal fibrogenesis, we examined the protein expression and location of UHRF1 in kidneys from two murine models of renal fibrosis (Fig. 1A, B). Compared with Sham group, both unilateral ureter obstruction (UUO) injury and unilateral renal ischemia-reperfusion (UIR) injury displayed increased renal UHRF1 protein expression (Fig. 1C, D). We next performed immunofluorescence double-labeling staining to assess UHRF1 expression in renal fibroblasts (labeled by anti-PDGFRα + β) or proximal tubular epithelial cells (labeled by anti-AQP1). It showed that UHRF1 protein either in fibroblasts or in proximal tubular epithelial cells was upregulated after UUO injury or UIR injury. Moreover, the number of UHRF1-labeled fibroblasts was more than UHRF1-labeled tubular epithelial cells (Fig. 1E, F). Taken together, these data suggest that UHRF1 expression is increased in fibrotic kidneys, mainly located in fibroblasts.

### UHRF1 is involved in fibroblasts activation

**Fig. 2:**
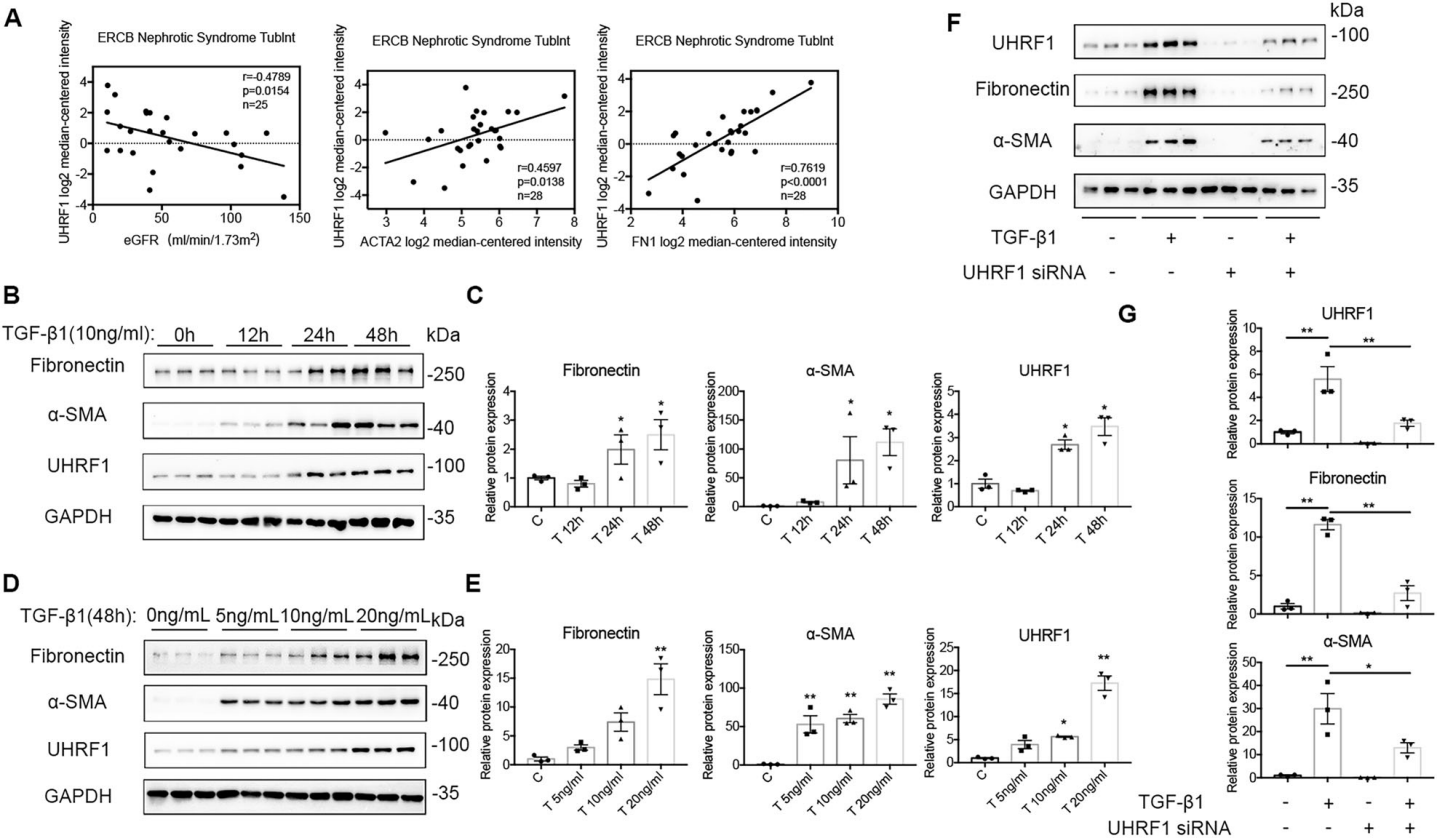
UHRF1 is involved in fibroblasts activation and renal fibrosis. **A** Correlation between UHRF1 expression and eGFR, ACTA2 expression and FN1 expression in a CKD cohort (ECRB Nephrotic Syndrome Tublnt). Data were analyzed using linear regression. **B**–**G** Relative protein expression of UHRF1, α-SMA and Fibronectin in NRK-49F cells, analyzed by western blots. NRK-49F cells were treated with 10 ng ml^−1^ TGF-β1 for 0 h, 12 h, 24 h, and 48 h respectively (**B**, **C**). NRK-49F cells were treated with different doses of TGF-β1 (0 ng ml^−1^, 5 ng ml^−1^, 10 ng ml^−1^, and 20 ng ml^−1^) for 48 h (**D**, **E**). NRK-49F cells were transfected with UHRF1 siRNA or control siRNA before treatment of 10 ng ml^−1^ TGF-β1 for 48 h (**F**, **G**). *n* = 3 samples per group. Data were shown as mean ± SEM. Data were analyzed using One-way ANOVA followed by Tukey. *P < 0.05, **P < 0.01.

To evaluate whether the increased expression of UHRF1 was associated with the progression of interstitial fibrosis, we first analyzed the correlation between UHRF1 mRNA expression and estimated glomerular filtration rate (eGFR), *ACTA2*(encoding α-SMA) mRNA expression, and *FN1*(encoding Fibronectin) mRNA expression in diabetic nephropathy (DN) and focal segmental glomerulosclerosis (FSGS) patients from ERCB Nephrotic Syndrome Tublnt Dataset using Nephroseq V5 online platform. We found UHRF1 expression was dramatically negatively correlated with eGFR, and positively correlated with fibrotic markers (*ACTA2* and *FN1*) expressions (Fig. 2A). Similarly, in vitro assays also shown that TGF-β1 dose- and time-dependently increased UHRF1 protein expression, accompanied by gradually increasing expressions of α-SMA and Fibronectin (Fig. 2B–E). Following by UHRF1 siRNA knockdown treatment, TGF-β1-induced fibroblasts activation was partially inhibited (Fig. 2F, G). These results indicated that upregulation of UHRF1 contributed to renal fibrosis and fibroblasts activation.

### Fibroblast-specific deletion of UHRF1 reduces renal fibrosis

**Fig. 3:**
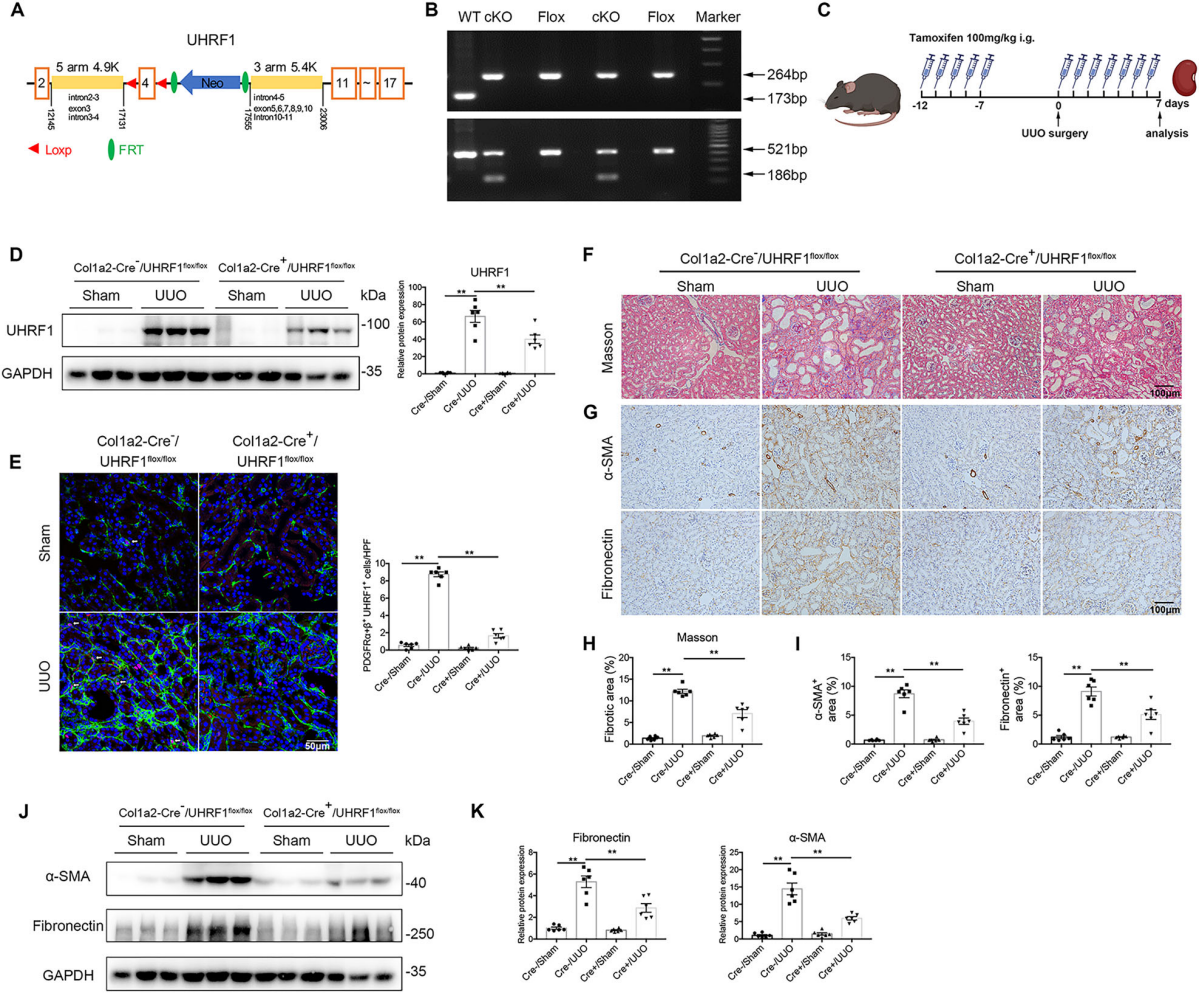
Fibroblast-specific depletion of UHRF1 inhibits UUO-induced renal fibrosis. Col1a2-Cre^+^/UHRF1^flox/flox^ mice (fibroblast-specific depletion of UHRF1) and their littermate controls received Sham or UUO operation for 7 days. **A** Generation of mice with fibroblast-specific depletion of UHRF1 using Cre-LoxP recombination system. **B** Genotyping of conditional knockout (cKO) mice (Col1a2-Cre^+^/UHRF1^flox/flox^ mice). **C** Illustration of experimental design to induce fibroblast-specific depletion of UHRF1 in UUO mice. **D** Relative renal UHRF1 protein expression analyzed by Western blots. **E** Immunofluorescence double-labeling with antibodies to UHRF1 (Red) and PDFGRα + β (green) (×200). The white arrow represents UHRF1^+^ fibroblasts. Scale bar: 50 μm. Masson staining (**F**, **H**) and immunochemistry staining for α-SMA and Fibronectin (**G**, **I**) in kidney sections (×200). Scale bar: 100 μm. (**J**, **K**) Western blots show the renal expression of α-SMA and Fibronectin. *n* = 6 mice per group. Data were shown as mean ± SEM. Data were analyzed using One-way ANOVA followed by Tukey. *P < 0.05, **P < 0.01.

**Fig. 4:**
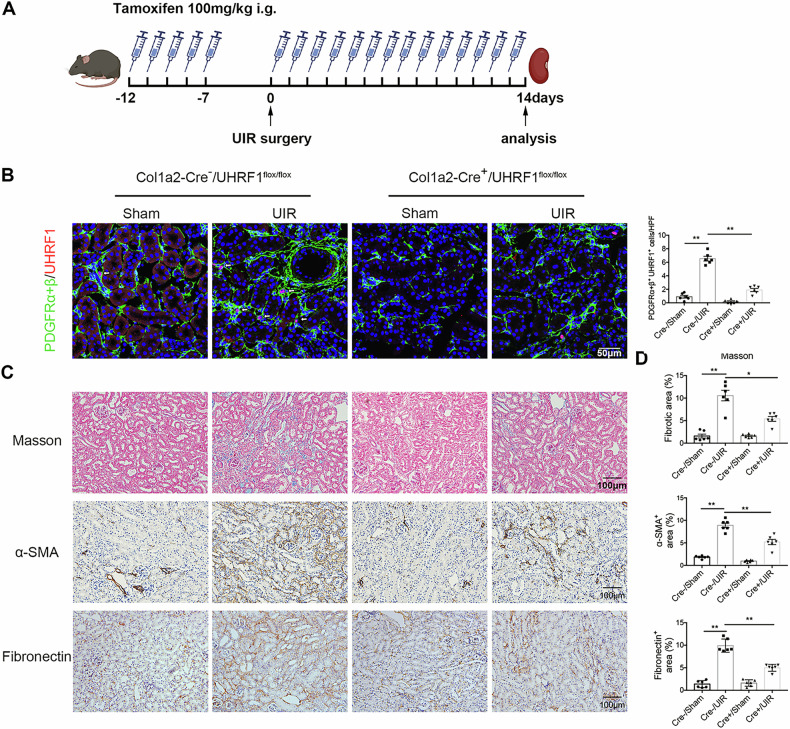
Fibroblast-specific depletion of UHRF1 inhibits UIR-induced renal fibrosis. Col1a2-Cre^+^/UHRF1^flox/flox^ mice (fibroblast-specific depletion of UHRF1) and their littermate controls received Sham or UIR operation for 14 days. **A** Illustration of experimental design to induce fibroblast-specific depletion of UHRF1 in UIR mice. **B** Immunofluorescence double-labeling with antibodies to UHRF1 (Red) and PDFGRα + β (green) (×200). The white arrow represents UHRF1^+^ fibroblasts. Scale bar: 50 μm. **C**, **D** Masson staining and immunochemistry staining for α-SMA and Fibronectin in kidney sections (×200). Scale bar: 100 μm. *n* = 6 mice per group. Data were shown as mean ± SEM. Data were analyzed using One-way ANOVA followed by Tukey. *P < 0.05, **P < 0.01.

To determine whether the observed function of UHRF1 on interstitial fibrosis was mediated by UHRF1 in fibroblasts, we constructed fibroblast-specific depletion of UHRF1 mice (Col1a2-Cre^+^ UHRF1^flox/flox^ mice) (Fig. 3A–C). These mice were then subjected to UUO or UIR surgery to induce renal fibrosis. As shown in Fig. 3D, renal UHRF1 protein was markedly decreased in Col1a2-Cre^+^/UHRF1^flox/flox^ mice compared with Col1a2-Cre^+^/UHRF1^flox/flox^ mice after UUO injury. Immunofluorescence double-labeling staining revealed that UHRF1 protein in fibroblasts was also significantly reduced in Col1a2-Cre^+^/UHRF1^flox/flox^ mice after UUO injury (Fig. 3E). Masson staining presented reduced collagen deposition in the obstructed kidneys of Col1a2-Cre^+^/UHRF1^flox/flox^ mice compared with littermate controls (Fig. 3F, H). Besides, fibroblast-specific depletion of UHRF1 attenuated the upregulation of α-SMA and Fibronectin protein in UUO-injured kidneys (Fig. 3G, I–K). In consistent with observations in UUO models, fibroblast-specific depletion of UHRF1 also reversed UIR-induced ECM accumulation and interstitial fibrosis (Fig. 4A–D).

### KLF15 in fibrotic kidney fibroblasts is hypermethylated and downregulated

**Fig. 5:**
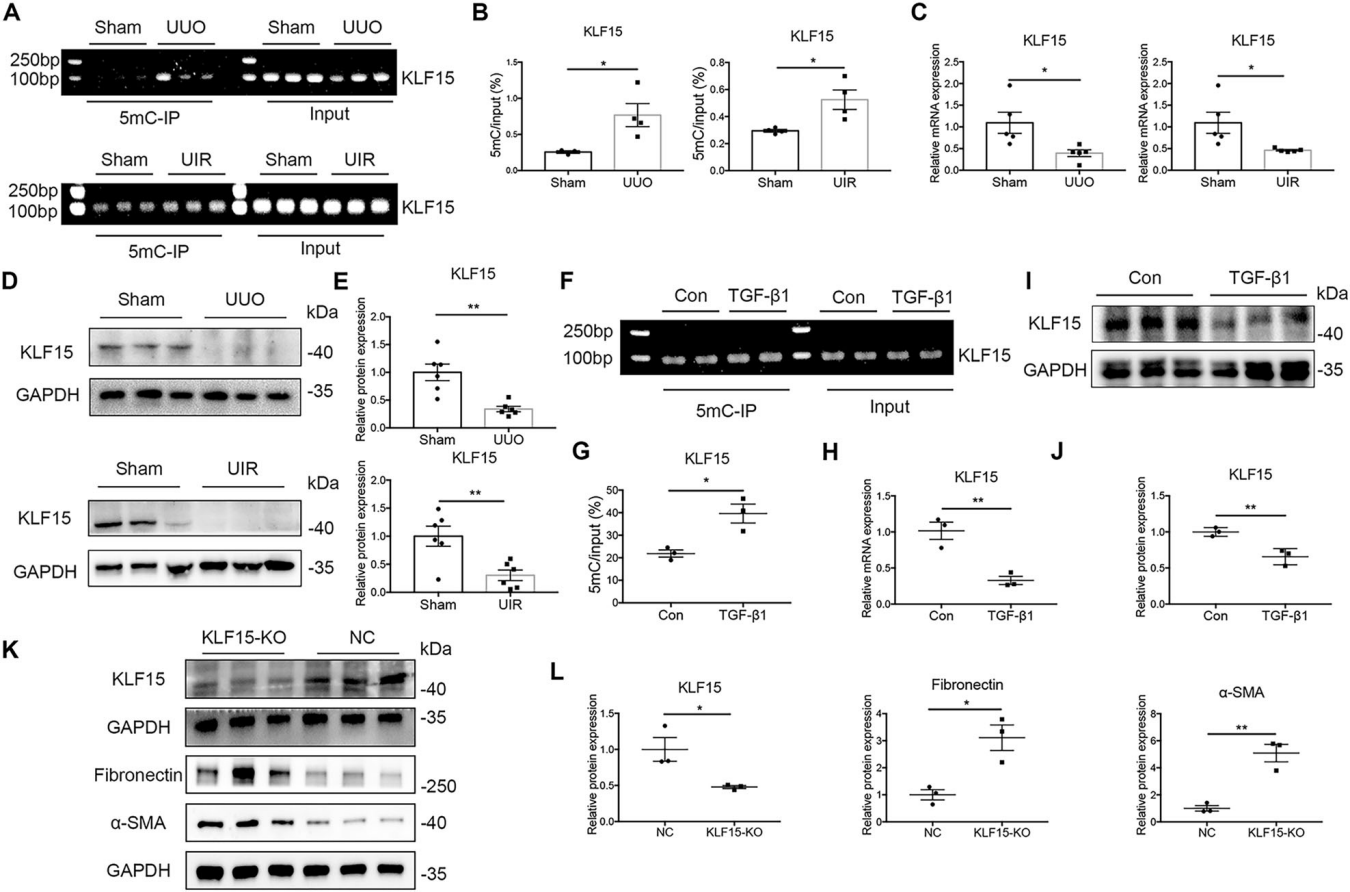
KLF15 hypermethylation in fibrotic kidneys and activated fibroblasts. **A**, **B** MeDIP-qPCR analysis shows the level of renal KLF15 promotor methylation in UUO mice or in UIR mice. The left panel shows a virtual gel of PCR products of methylated DNA and input DNA. The right panel shows quantitative real-time PCR results. *n* = 4 mice per group. **C** Relative renal KLF15 mRNA expression in UUO mice or in UIR mice, analyzed by qRT-PCR. *n* = 5 mice per group. **D**, **E** Relative renal KLF15 protein expression in UUO mice or in UIR mice, analyzed by Western blots. *n* = 6 mice per group. **F**–**J** NRK-49F cells were treated with 10 ng/ml TGF-β1 for 48 h. And KLF15 promotor methylation was analyzed by MeDIP-qPCR (**F**, **G**). Relative KLF15 mRNA expression, analyzed by qRT-PCR (**H**). Relative KLF15 protein expression, analyzed by Western blots (**I**, **J**). **K**, **L** NRK-49F cells were infected with CRISPR-Cas9 lentivirus targeting KLF15. Relative KLF15, α-SMA and Fibronectin protein expression were analyzed by Western blots. *n* = 3 samples per group. Data were shown as mean ± SEM. Data were analyzed using unpaired Student’s *t* test. *P < 0.05, **P < 0.01.

We next aimed to explore the mechanisms underlying UHRF1 in progression of renal fibrosis. To characterize the altered DNA methylation profiles in activated renal fibroblasts, renal fibroblasts were sorted out by FCAS from Sham mice or UUO mice. Genomic DNA was extracted from these cells, and methylation profile was performed using RRBS. A total of 26,996 DMRs (17333 hypermethylated and 9663 hypomethylated) on antosomal chromosomes were identified in UUO mice versus Sham mice (Fig. S1A, Supporting Information). KEGG pathway analysis revealed that DMRs were enriched in Wnt signaling pathway, Ras signaling pathway, MAPK signaling pathway, all of which are involved in progression of renal fibrosis (Fig. S1B, Supporting Information). Among these hypermethylated DMRs, we identified the top 50 genes based on adjusted *P* values and methylation level differences (Table S1, Supporting Information). While among these genes, only KLF15 has been confirmed by previous studies to be involved in renal fibroblast activation [19, 20], which makes it a candidate to be studied in epigenetic mechanism of kidney fibrosis. To gain insights into the role of KLF15 hypermethylation (Fig. S1C, Supporting Information) on renal fibrosis, we investigated its methylation and expression both in vivo and in vitro. MeDIP analysis revealed that renal KLF15 was hypermethylated in the two mouse models (Fig. 5A, B). Besides, mRNA and protein expression of renal KLF15 were decreased both in UUO and UIR models (Fig. 5C–E), confirming that KLF15 hypermethylation correlates with decreased KLF15 expression. In vitro, NRK-49F cells were treated with TGF-β1 to activate fibroblasts. Similarly, KLF15 was hypermethylated and downregulated in activated fibroblasts (Fig. 5F–J). Besides, KLF15 knockdown by CRISPR-Cas9 system also resulted in activation of fibroblasts, manifested by upregualtion of α-SMA and Fibronectin (Fig. 5K, L). Taken together, these results suggested that KLF15 hypermethylation-induced loss of KLF15 accounted for fibroblasts activation and renal fibrosis.

### UHRF1 contributes to KLF15 hypermethylation and loss of KLF15 protein

**Fig. 6:**
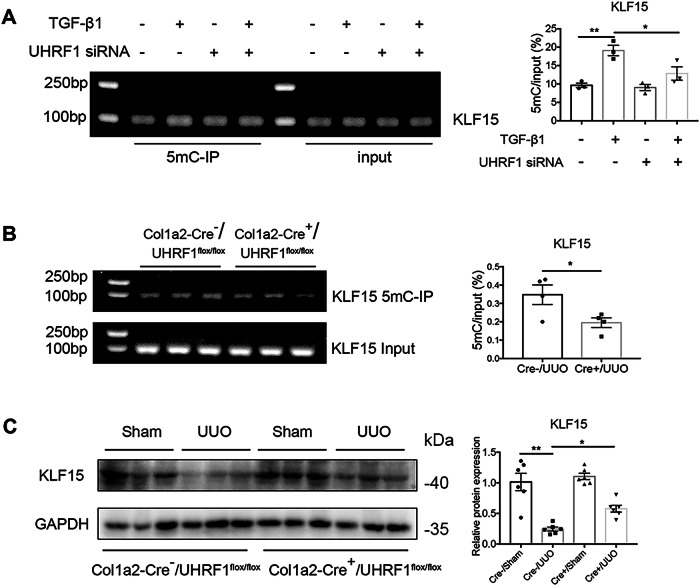
Knockdown of UHRF1 inhibits fibroblasts activation by demethylating KLF15 and restoring its expression. **A** MeDIP-qPCR analysis shows the level of KLF15 promotor methylation. NRK-49F cells were transfected with UHRF1 siRNA or control siRNA before treatment of 10 ng/ml TGF-β1 for 48 h. *n* = 3 samples per group. **B**, **C** Col1a2-Cre^+^/UHRF1^flox/flox^ mice (fibroblast-specific depletion of UHRF1) and their littermate controls received Sham or UUO operation for 7 days. **B** Renal KLF15 promotor methylation levels were determined by MeDIP-qPCR. *n* = 4 mice per group. **C** Renal KLF15 protein levels were determined by Western blots. *n* = 6 mice per group. Data were shown as mean ± SEM. Data were analyzed using unpaired Student’s *t* test (**B**) or One-way ANOVA followed by Tukey (**A**, **C**). *P < 0.05, **P < 0.01.

Considering the role of UHRF1 in DNA methylation, we performed MeDIP analysis to assess whether knockdown of UHRF1 influences KLF15 methylation. The results showed that UHRF1 knockdown reversed TGF-β1-induced KLF15 hypermethylation (Fig. 6A). And in vivo, fibroblast-specific depletion of UHRF1 ameliorated UUO-induced KLF15 hypermethylation and decreased KLF15 protein (Fig. 6B, C).

### Interaction between UHRF1 and DNMT1 mediates KLF15 hypermethylation and fibroblasts activation

**Fig. 7:**
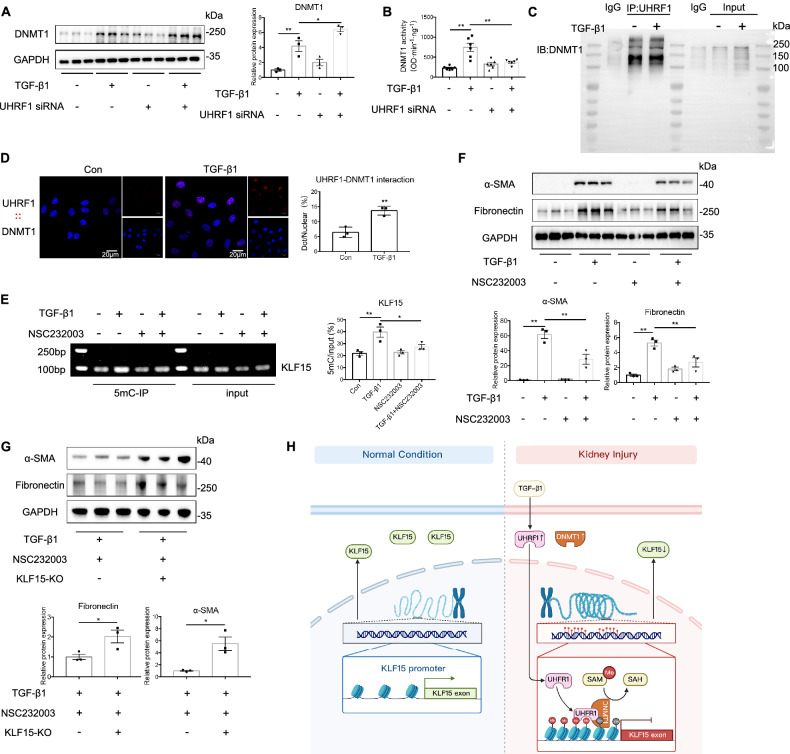
Interaction between UHRF1 and DNMT1 mediates KLF15 hypermethylation and fibroblasts activation. **A** Western blots showed the DNMT1 expression in NRK-49F cells transfected with UHRF1 siRNA or control siRNA with or without TGF-β1 treatment. **B** DNMT1 enzyme activity of NRK-49F cells transfected with UHRF1 siRNA or control siRNA with or without TGF-β1 treatment. **C** Co-IP analysis showed anti-UHRF1 antibody pulled down endogenous DNMT1. **D** PLA assay showed the interaction between UHRF1 and DNMT1 in NRK-49F cells with or without TGF-β1 treatment, presented by red fluorenscent signals (×400). Scale bar: 20 μm. **E** MeDIP-qPCR analysis showed inhibition of the interaction between UHRF1 and DNMT1 with NSC232003 prevented TGF-β1-induced KLF15 hypermethylation in NRK-49F cells. **F** Western blots demonstrated blocking effect of NSC232003 on TGF-β1-induced fibroblasts activation in NRK-49F cells. **G** NRK-49F cells infected with CRISPR-Cas9 lentivirus targeting KLF15 or scramble lentivirus were treated with TGF-β1 and NSC232003. Fibroblasts activation was assessed by immunoblots of α-SMA and Fibronectin. *n* = 3–6 samples per group. Data were shown as mean ± SEM. Data were analyzed using unpaired Student’s *t* test (**D**, **G**) or One-way ANOVA followed by Tukey (**A**, **B**, **E**, **F**). *P < 0.05, **P < 0.01.

We next aimed to explore the molecular mechanism underlying UHRF1-induced KLF15 hypermethylation. Since DNA methylation is directly mediated by DNMT1, we first examined DNMT1 expression. Compared with Sham operation, renal DNMT1 protein expression was increased after UUO or UIR injury (Fig. S2A, B, Supporting Information). Additionally, TGF-β1 enhanced DNMT1 expression in fibroblasts in the same manner (Fig. S2C, Supporting Information). However, UHRF1 knockdown failed to downregulated DNMT1 expression (Fig. 7A), which suggested UHRF1-induced KLF15 hypermethylation was not achieved through upregulating DNMT1 expression. In addition to protein expression, DNMT1 activity can be modulated by molecular interactions. Interestingly, we found TGF-β1 enhanced DNMT1 activity, which was inhibited following UHRF1 knockdown (Fig. 7B). We then examined the interaction between UHRF1 and DNMT1 in renal fibroblasts. Coimmunoprecipitation (Co-IP) assay suggested the interaction between UHRF1 and DNMT1 in NRK-49F cells, which was further confirmed by Proximity ligation assay (PLA) (Fig. 7C, D). Furthermore, PLA assay showed that TGF-β1 enhanced the interaction between UHRF1 and DNMT1. To further evaluate the role of such interaction on KLF15 methylation, we treated NRK-49F cells with NSC232003, a small molecule inhibitor by inhibiting the interaction between UHRF1 and DNMT1 [21]. Western blot and PLA assay also verified that NSC232003 weakened such interaction without downregulating expression of UHRF1 and DNMT1 (Fig. S3A, B, Supporting Information). We observed that NSC232003 significantly decreased TGF-β1-induced KLF15 hypermethylation, together with lower expression of α-SMA and Fibronectin (Fig. 7E, F), which was abolished by suppression of KLF15 using CRISPR-Cas9 editing system (Fig. 7G). Such data implied that UHRF1 modulated KLF15 methylation and fibroblasts activation dependent on the interaction with DNMT1.

## Discussion

DNMT1-mediated DNA maintenance methylation plays an important role in the progression of renal fibrosis. Although increasing evidence indicates UHRF1 assists DNMT1 in maintaining DNA methylation [13, 14, 16, 22, 23], current understanding of its pathological function in renal fibrosis is unclear. In this study, we observed a significant upregulation of UHRF1 in fibrotic kidneys from two murine models of renal fibrosis. Further immunofluorescence double-labeling staining determined upregulated UHRF1 was mainly located in fibroblasts. Renal fibrosis is a pathological wound healing in response to injury, featured by excessive extracellular matrix deposition and proliferation of fibroblasts. Apart from an epigenetic coordinator, UHRF1 was firstly recognized as a regulator of the cell cycle, mainly expressed in proliferating cells rather than highly differentiated cells [24, 25]. Numerous studies revealed that overexpression of UHRF1 was frequently found in various cancers and contributed to poor prognosis partly by promoting cell proliferation [26–28]. UHRF1 was also reported to participate in airway tissue repair by stimulating stem cell proliferation [29]. Similarly, our research revealed UHRF1 expression preferred actively proliferative fibroblasts to low proliferative proximal tubular epithelial cells. Fatherly, its expression was positively correlated with activation of fibroblasts induced by TGF-β1. Conversely, fibroblast-specific depletion of UHRF1 alleviated activation of fibroblasts or renal fibrosis both in vivo and in vitro. A recent study also reported knockdown of UHRF1 resulted in arrested lung fibroblasts proliferation and lung fibrosis [30]. All of these indicated UHRF1 was essential for renal fibrogenesis.

Altered DNA methylation profiles have been observed both in multiple animal models of renal fibrosis and in renal biopsies from CKD patients [3, 31–35]. However, recent studies on DNA methylation sequencing in fibrotic kidneys, which is limited by the heterogeneity of cell types in kidney cortices, fail to reveal fibroblast-specific DNA methylation profiles. Therefore, we performed RRBS on renal fibroblasts isolated from UUO mice using flow sorting. We found different methylated regions were enriched in renal fibrosis-associated pathway including Wnt signaling pathway, Ras signaling pathyway, MAPK signaling pathway and so on.

Interestingly, we found KLF15 hypermethylation in activated renal fibroblasts from sequencing data, which was testified by MeDIP-qPCR both in vivo and in vitro. Considering that promotor methylation inhibits gene transcription, we also examined expressions of KLF15 mRNA and protein. Consistent with previous findings, KLF15 was downregulated in activated renal fibroblasts and fibrotic kidneys [19, 20, 36–38]. KLF15 is expressed in fibroblasts, podocytes, mesangial cells, endothelial cells and proximal tubular epithelial cells in kidneys [39]. As a transcriptional regulator, KLF15 exerts multiple biological functions such as anti-fibrosis effect, regulation of gluconeogenesis and adipocyte differentiation [19, 40, 41]. And in renal pathophysiological processes, KLF15 was reported to be involved in podocyte differentiation, mesangial proliferation, inflammatory response and renal fibrosis [19, 42–50]. Previous studies indicated inhibiting of KLF15 would exacerbate renal myofibroblasts proliferation and extracellular matrix deposition [19], similar to our in vitro findings. The underlying mechanisms of its antifibrotic role are associated with inhibition of canonical Wnt/β-catenin pathway, suppression of CTGF expression and restoring loss of fatty acid oxidation [19, 20, 38]. Hence, hypermethylation of KLF15 not only explains its loss in fibrotic kidneys, but also results in progress of renal fibrosis.

Since maintaining DNA methylation is the most studied epigenetic functions of UHRF1, whether KLF15 was the downstream target gene of UHRF1 and its methylation pattern was modulated by UHRF1 would be explored. Surprisingly, we found knockdown of UHRF1 could reverse TGF-β1-induced KLF15 hypermethylation, accompanied by restoring expression of KLF15. However, DNMT1 expression was not influenced by UHRF1 knockdown. Bostick et al. also confirmed that loss of methylation pattern by depletion of UHRF1 in embryonic stem cells was not arisen from decreases in DNA methyltransferases [13]. Instead, DNMT1 enzyme activity was inhibited after UHRF1 knockdown in our research. It was reported that the RFTS domain of DNMT1 inhibited DNA-binding and catalytic activity of DNMT1 through plugging the catalytic pocket [51]. However, UHRF1 could remove the RFTS from the catalytic pocket of DNMT1 to enhance DNMT1 enzyme activity via the interaction between its SRA domain and RFTS [52]. Consistently, we observed the interaction between UHRF1 and DNMT1, and TGF-β1 enhanced this interaction. Moreover, inhibition of this interaction by NSC232003, evidenced by PLA assay, reversed hypermethylation and loss of KLF15 induced by TGF-β1, leading consequently to reduced fibroblast activation, while knockdown of KLF15 abolished such role played by NSC232003.

In summary, we for the first time demonstrated that UHRF1 was upregulated in activated renal fibroblasts and it was a critical epigenetic regulator for progression of renal fibrosis, utilizing both genetic depletion or pharmacological approaches. Mechanically, UHRF1 maintained KLF15 promotor hypermethylation through interacting with DNMT1, resulting in loss of KLF15 and fibroblasts activation. Our study suggests that UHRF1 is a promising therapeutic target for renal fibrosis.

## Materials and methods

### Mice kidney fibrotic models

Six- to eight-week-old male C57BL/6 wild-type (WT) mice were purchased from Shanghai Model Organisms Center and housed in specific pathogen-free conditions, free to water and diet. Mice were randomly assigned to Sham group or experiment group. For experiment group, mice were subjected to UUO or UIR surgery to induce renal fibrosis, followed by anesthesia using pentobarbital sodium. UUO was performed by ligation of the right ureter with 4-0 silk for seven days. To induce UIR, right renal pedicles were clamped with microaneurysm clips for 35 min with maintaining body temperature at 38 °C. Injured kidneys were harvested 2 weeks after UIR operation. For Sham group, right kidneys were only exposed without treatment.

### Generation of fibroblast-specific UHRF1 conditional knockout mice

UHRF1^flox/flox^ mice and Col1a2-Cre/ERT2 transgenic mice were purchased from Shanghai Model Organisms Center. To generate the inducible fibroblast-specific UHRF1 conditional knockout mouse lines, UHRF1^flox/flox^ mice were bred with Col1a2-Cre/ERT2 transgenic mice. Six- to eight-week-old male Col1a2-Cre^+^ UHRF1^flox/flox^ (cKO) mice and Col1a2-Cre^-^ UHRF1^flox/flox^ mice were randomly assigned to receive Sham or UUO/UIR operation. To activate the Cre-ERT system, homozygous male mice (Col1a2-Cre^+^ UHRF1^flox/flox^) were treated with 100 mg kg^−1^ tamoxifen (Sigma) via oral gavage for 5 consecutive days one week before operation. After Sham or UUO/UIR operation, daily gavage of tamoxifen was continued until the day before sacrifice. Col1a2-Cre^-^ UHRF1^flox/flox^ mice received the same dose of tamoxifen. Only the investigators who conducted animal operations were aware of group allocation.

### Renal pathological staining

Masson trichrome staining and immunohistochemistry staining were performed as previously described [32]. Primary antibodies used in this study were anti-α-SMA (Sigma, A2547) and anti-Fibronectin (Abcam, ab2413). Images were taken with a light microscopy. Ten fields of each sample were randomly selected and analyzed using Image J software.

### Immunofluorescence

Tissue sections of 4 μm in thickness were utilized for immunofluorescence staining. Primary antibodies used in this study were anti-UHRF1 (Santa Cruz Biotechnology, sc373750), anti-PDGFRα + β (Abcam, ab32570), and anti-AQP1 (Abcam, ab168387). The nuclei were stained with Hochest 33342. Images were taken with Olympus FV3000 confocal microscope. Specific cells in renal sections were counted in 10 randomly selected cortical interstitial fields per mouse.

### Cell culture and treatment

Rat normal renal fibroblast (NRK-49F cells, ATCC) were cultured in DMEM supplemented with 10% fetal bovine serum (FBS) and 1% penicillin and streptomycin. NRK-49F cells were starved overnight in DMEM containing 0% FBS prior to any stimulation. Pre-incubation of 50 μM NSC232003 (MCE) was carried out 1 h before TGF-β1 (RD) treatment. The dosage levels and times of TGF-β1 were adopted as indicated. Cells were free from mycoplasma contamination.

### siRNA transfection

NRK-49F cells were plated in 12-well culture dishes and transfected the next day (50% confluent) with UHRF1 siRNA (target sequence: TCATGTACCACATCAAGTA) or negative control siRNA using lipofectamine 3000 (Invitrogen) according to the manufacturer’s instructions.

### Western blotting

Renal tissues or cells were lysed in RIPA lysis buffer containing PMSF. Primary antibodies used in this study were anti-UHRF1 (Santa Cruz Biotechnology), anti-α-SMA (Sigma), anti-Fibronectin (Abcam), anti-GAPDH (Proteintech, 60004-1-Ig), anti-DNMT1 (Cell Signaling Technology, 5032), and anti-KLF15 (Abcam, ab2647). The intensity of bands was quantified using Image J software.

### RNA isolation and real-time RT-PCR

RNA isolation and qRT-PCR were performed as previously described [32]. The mRNA levels were normalized for the level of GAPDH. Specific oligonucleotide primers were listed in Supplementary Table S2.

### MeDIP-qPCR

Genomic DNA sample was extracted from tissues or cells using TIANamp Genomic DNA Kit (TIANGEN) according to the manufacturer’s instructions. MeDIP was performed as described previously [53]. After purification by using MinElute PCR Purification Kit (Qiagen), methylated DNA was amplificated using SYBR Premix Ex Taq Kit (TaKaRa) and 7500 Real-time PCR System (Applied Biosystems, MA, USA). The results were shown as percentage of input (%input). PCR products were electrophoresed and imaged by Gel Imaging System (Tanon, Shanghai, China). Gene-specific primers were listed in Supplementary Table S3.

### Coimmunoprecipitation (Co-IP)

Cells were harvested in IP Lysis buffer containing cocktail proteinase and PMSF. Anti-UHRF1 antibody (Santa Cruz Biotechnology) and protein A/G magnetic beads (Thermo Fisher Scientific) were used for immunoprecipitation according to manufacturer’s protocols. UHRF1-combined proteins were analyzed by Western blot. IgG was used as a negative control.

### Proximity ligation assay (PLA)

PLA was performed with in situ proximity ligation assay (Sigma) kit according to manufacturer’s protocols. Interaction between UHRF1 and DNMT1 were detected by PLA signals (red fluorescent) imaged by conventional and confocal fluorescent microscope.

### Renal fibroblasts sorting and Reduced Representation Bisulfite Sequencing (RRBS)

Mouse renal fibroblasts were acquired as previously reported [54]. Briefly, kidneys were shredded and digested by 1 mg ml^−1^ Collagenase I (Thermo Fisher Scientific), followed by dissociation by Miltenyi gentleMACS Dissociator. After removal of red blood cells and blocking by anti-mouse CD16/32 antibodies (Biolegend, 156603), a single cell suspension was incubated with FITC-CD45 antibodies (Biolegend, 103107), PE-CD31 antibodies (Biolegend, 102507) and APC-CD140a antibodies (Biolegend, 135907). Stained cells were sorted using FACS Aria II cell sorter (BD). Genomic DNA was extracted from FACS-sorted fibroblasts using TIANamp Micro DNA Kit (TIANGEN) and analyzed on RRBS by Cloud-Seq Biotech (Shanghai, China).

### CRISPR-Cas9 genomic deletion

The CRISPR-Cas9 lentivirus targeting KLF15 was constructed by Genechem (Shanghai, China). The gene sequence for generating sgRNA targeting *KLF15* was 5’-TCGCCCATCTCCGAGGATGA-3’. NRK-49F cells were selected with puromycin after infection and verified by RT-PCR and western blot assays.

### Statistics

All data were presented as mean ± SEM. Statistical analyses were carried out using GraphPad Prism software (version 7.0). For analyzing differences between two groups, Student’s *t* test was performed for the data normally distributed. For the statistical significance among multiple groups, One-way ANOVA followed by Dunnett test was conducted for the data with homogeneity of variance. *P* values (two-sided) <0.05 were considered statistically significant. Sample sizes were determined according to previous publications without prior power analysis.

## Supplementary information

supplemental material

Original western blots

## Data Availability

The entire RRBS dataset was available from Gene Expression Omnibus database (accession number GSE283271). Other supporting information is available from the corresponding author upon reasonable request.
