# Supplementary material for: Inhibition of epigenetic regulator UHRF1 attenuates renal fibrosis and retains transcription factor Krüppel-like factor 15 expression
Source: Cell Death Discov. 2025 Jun 9;11:270. doi: 10.1038/s41420-025-02549-y (PMC12149316; doi:10.1038/s41420-025-02549-y)
Supplement: Supplementary file 1 — supplemental material [file 41420_2025_2549_MOESM1_ESM.docx]

**Supplementary Figures**


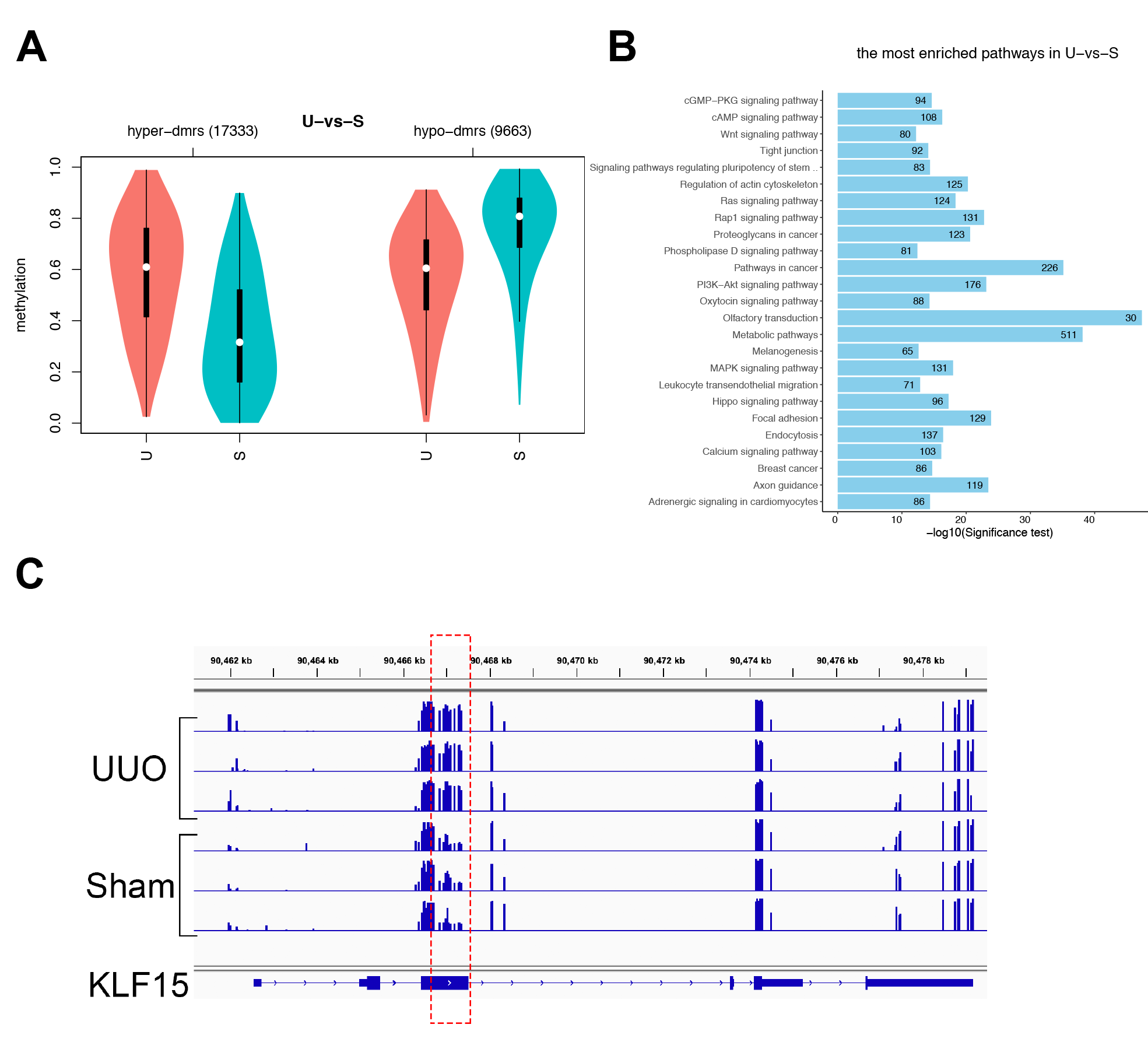


**Figure S1 DNA methylation profile of renal fibroblasts after UUO injury.**

(A) Violin plot of differentially methylated regions in UUO mice compared with Sham mice. (B) KEGG pathway enrichment analysis of differentially methylated genes. (C) Integrated Genomics Viewer displayed DNA methylation patterns of KLF15 in Sham and UUO mice.


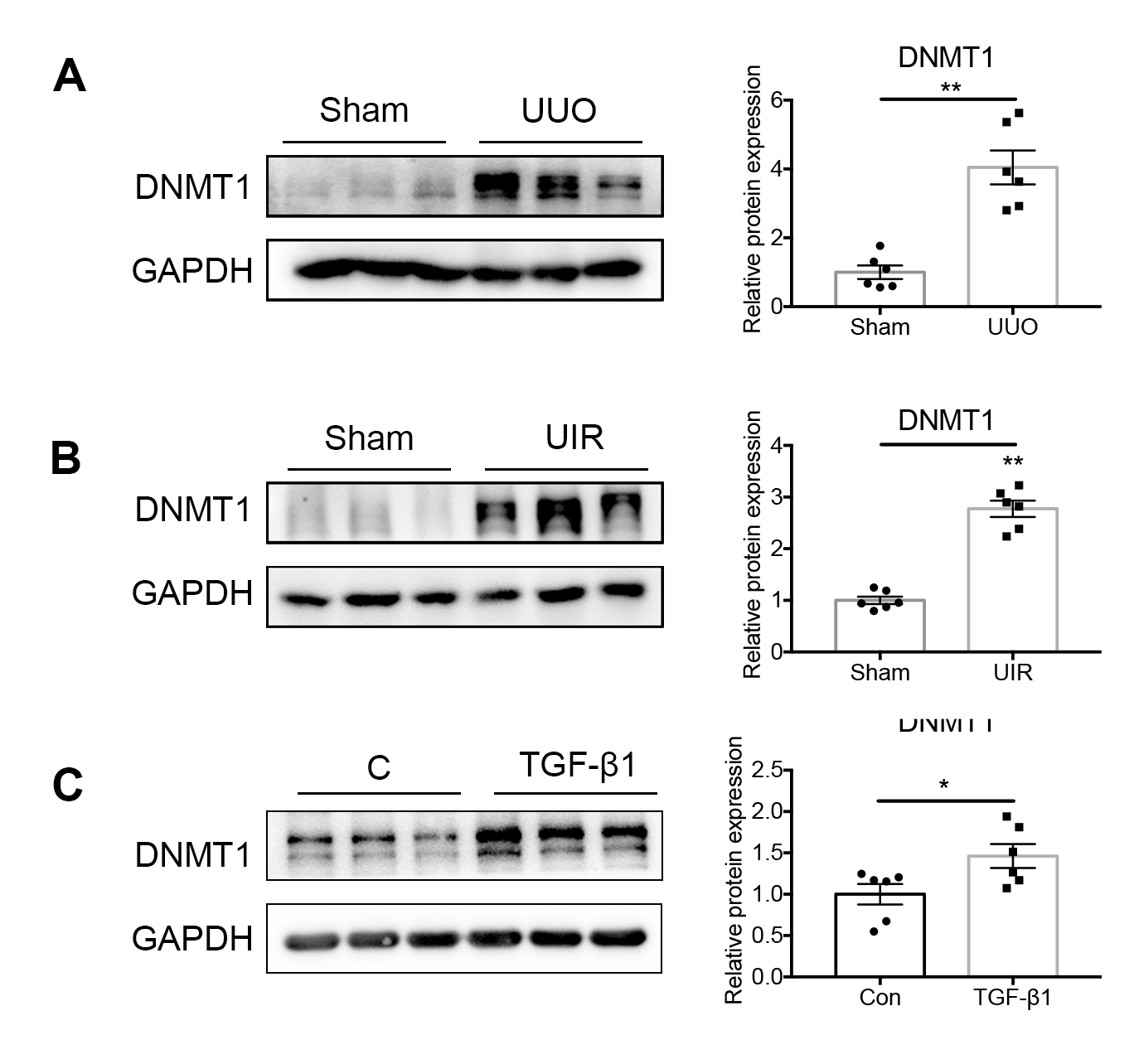


**Figure S2 Upregulation of DNMT1 in fibrotic kidneys and activated fibroblasts.**

(A, B) Renal DNMT1 protein expression was examined by western blots on days 7 in UUO mice and on days 14 in UIR mice. (C) DNMT1 expression of NRK-49F cells with or without TGF-β1 stimulation. *P<0.05, **P<0.01.


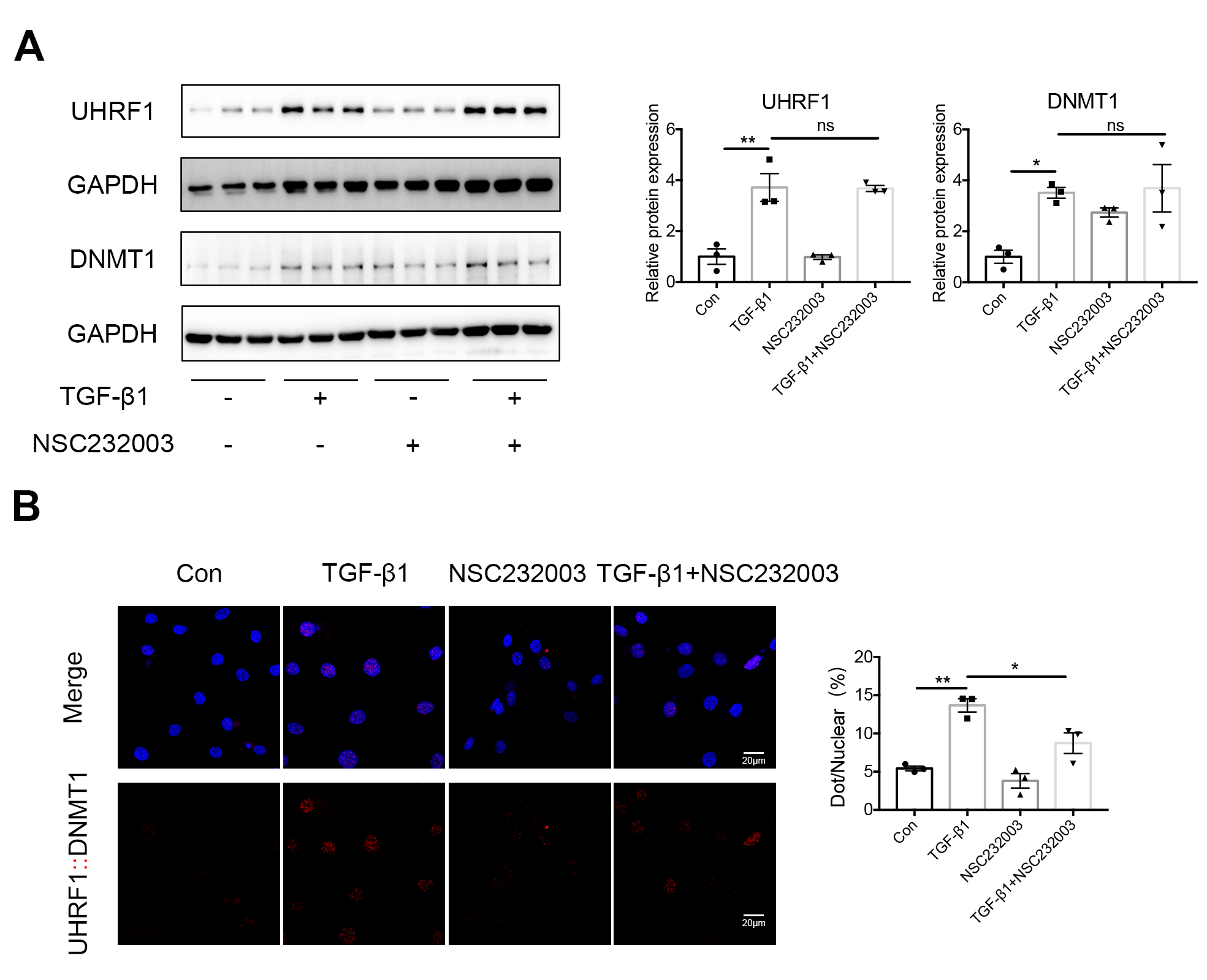


**Figure S3 NSC232003 inhibited interaction between UHRF1 and DNMT1.**

NRK-49F cells were treated with TGF-β1 or NSC232003 for 48 hours. (A) DNMT1 protein expression was determined by Western blots. (B) Interaction between UHRF1 and DNMT1 was determined by PLA. Protein interaction was shown by red fluorescence. Scale bar: 20 μm. *P<0.05, **P<0.01.

**Supplementary Tables**

**Table S1. List of top 50 hypermethylated genes in fibrotic mouse renal fibroblasts.**

| Slc25a25 | Klhdc7a | Vasn | Rnf186 | Clic5 | Wdr66 | Fam195a |
| --- | --- | --- | --- | --- | --- | --- |
| Arhgap23 | Hoxa5 | Anpep | Fbn2 | Nbeal2 | Slc23a1 | E2f1 |
| Hoxb3 | Hnf4a | Nuak2 | Prss8 | Shank3 | Tnfaip6 |  |
| Mn1 | Plekhg3 | Tbc1d16 | Arhgef18 | Ccm2l | Tbxa2r |  |
| Tenm3 | Slc11a2 | Ifrd2 | Myo18a | Plekhh1 | Rasal3 |  |
| Slc25a44 | Pxn | Zfpm1 | Hps1 | Sipa1 | Miox |  |
| Sbf1 | Tmem174 | Arfgef2 | Uvrag | Micall2 | Plekhg5 |  |
| Kif1c | Fbxo40 | Srsf6 | Inpp5b | **Klf15** | Ankrd13b |  |

**Table S2. Sequence of primers for RT-PCR**

| Gene | Forward primer | Reversed primer |
| --- | --- | --- |
| mouse KLF15 | GAGGTGGCTGCTCTTGGTGTACATC | CGTCCCGTAGACAAAATGG |
| mouse GAPDH | CGTCCCGTAGACAAAATGG | TCAATGAAGGGGTCGTTGA |
| Rat KLF15 | CCAAGAGCAGCCACCTCAAG | TCGCATACGGGACACTGGTA |
| Rat GAPDH | AGGTCGGTGTGAACGGATTTG | TGTAGACCATGTAGTTGAGGTCA |

**Table S3. Sequence of primers for MeDIP-qPCR**

| Gene | Forward primer | Reversed primer |
| --- | --- | --- |
| mouse KLF15 | GTCTGCACTTCGGGACTCTG | TCCGGTCTCTTCGTGGATCT |
| Rat KLF15 | CGAGATCCTACGTGGTCTGC | TGCGCACGTGAGATAGGATC |
