# Supplementary material for: Inhibition of epigenetic regulator UHRF1 attenuates renal fibrosis and retains transcription factor Krüppel-like factor 15 expression
Source: Cell Death Discov. 2025 Jun 9;11:270. doi: 10.1038/s41420-025-02549-y (PMC12149316; doi:10.1038/s41420-025-02549-y)
Supplement: Supplementary file 2 — Original western blots [file 41420_2025_2549_MOESM2_ESM.pdf]

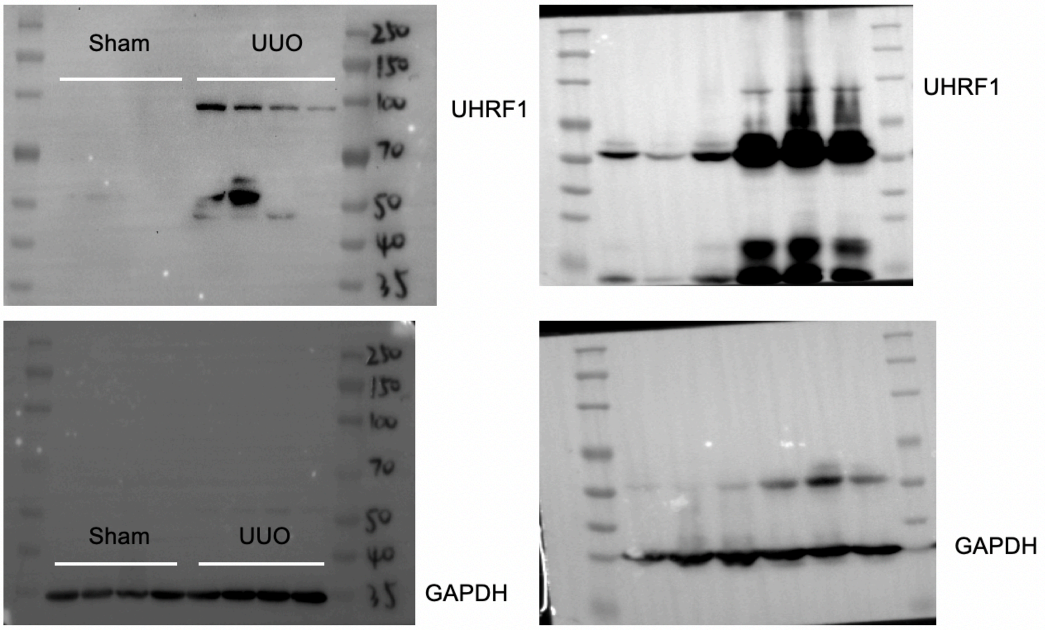


**Figure 1C**

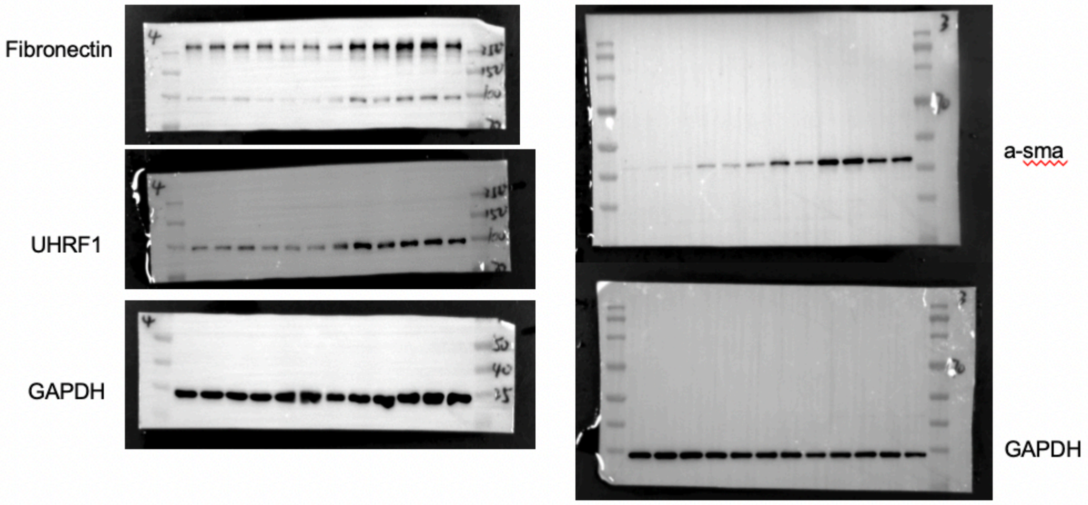


**Figure 2B**

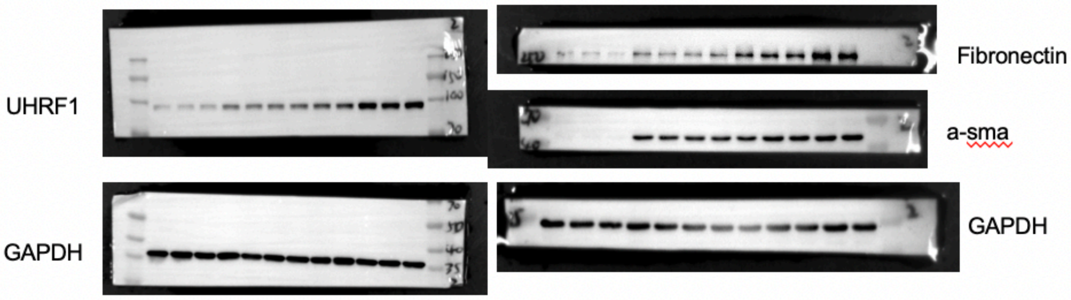


**Figure 2D**

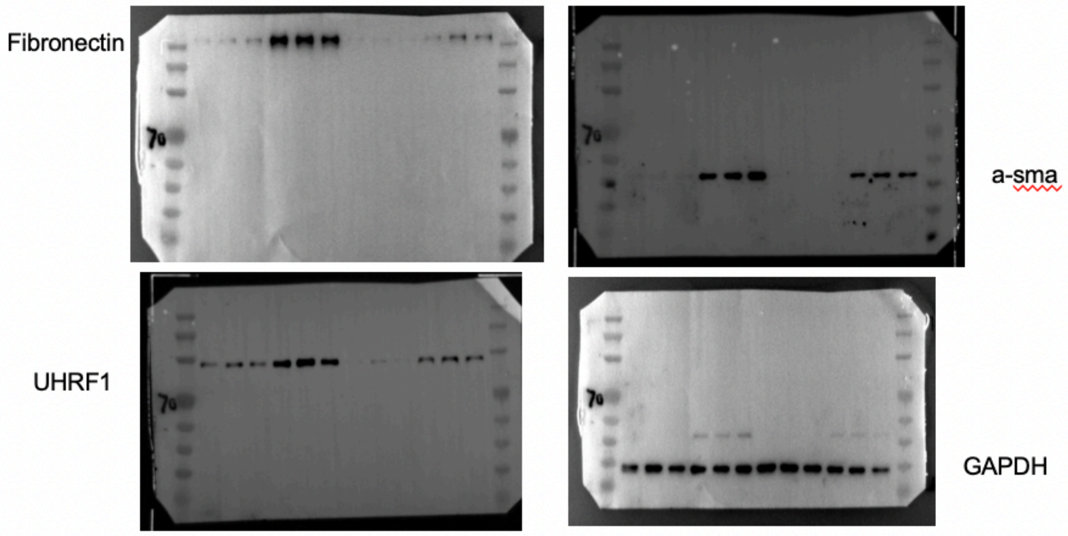


**Figure 2F**

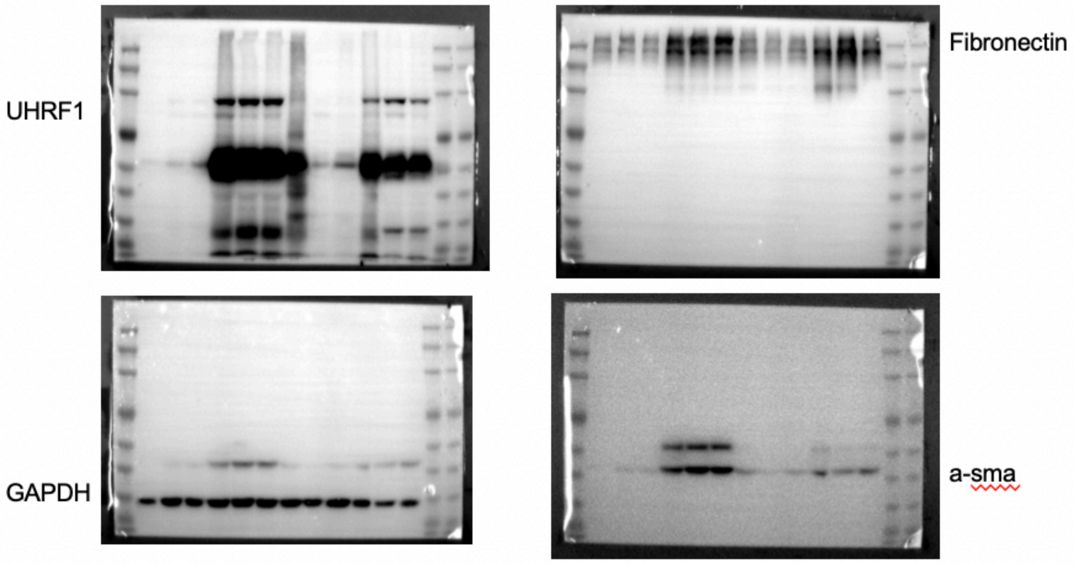


**Figure 3D、J**

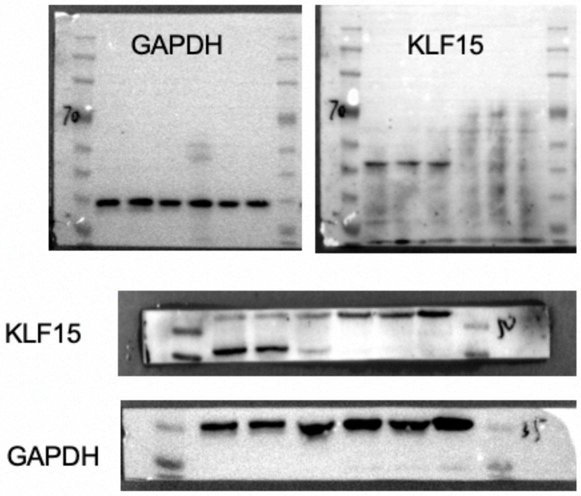


**Figure 5D**

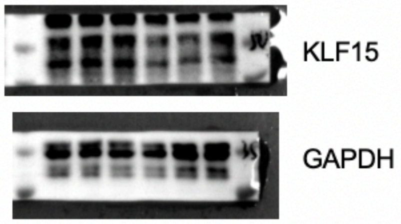


**Figure 5I**

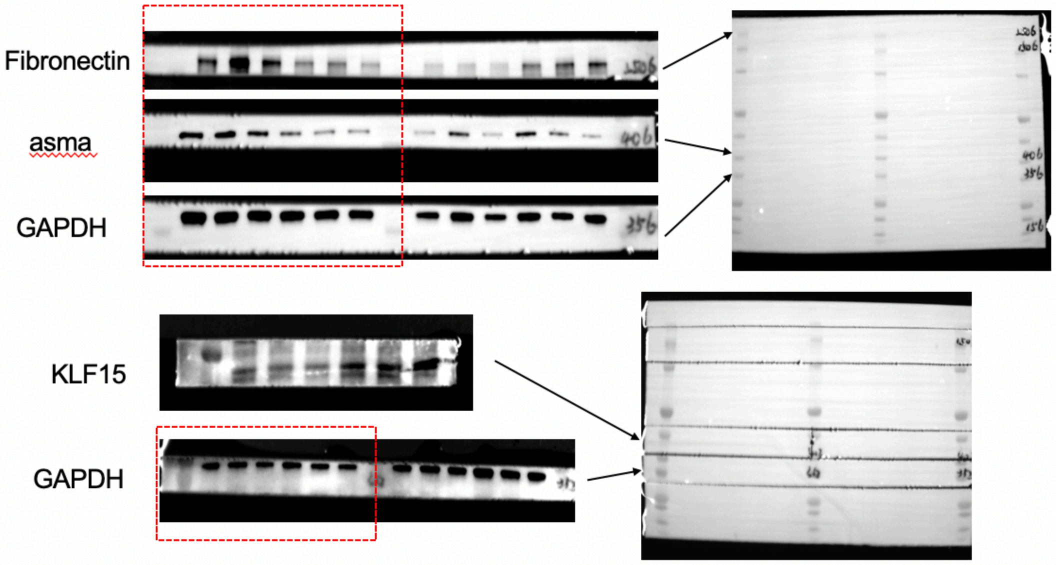


**Figure 5K**

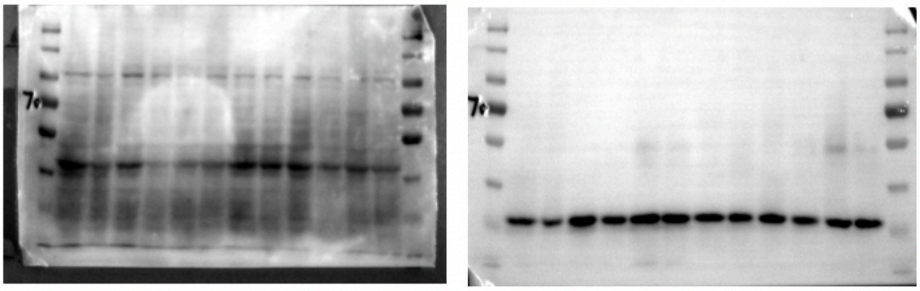

KLF15 GAPDH

**Figure 6C**

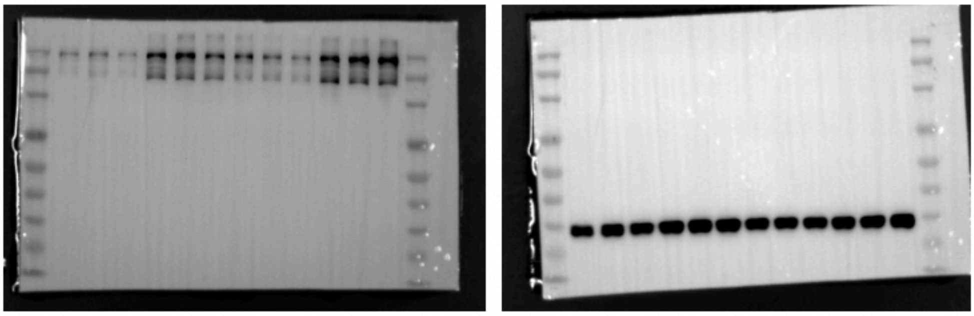

DNMT1 GAPDH

**Figure 7A**

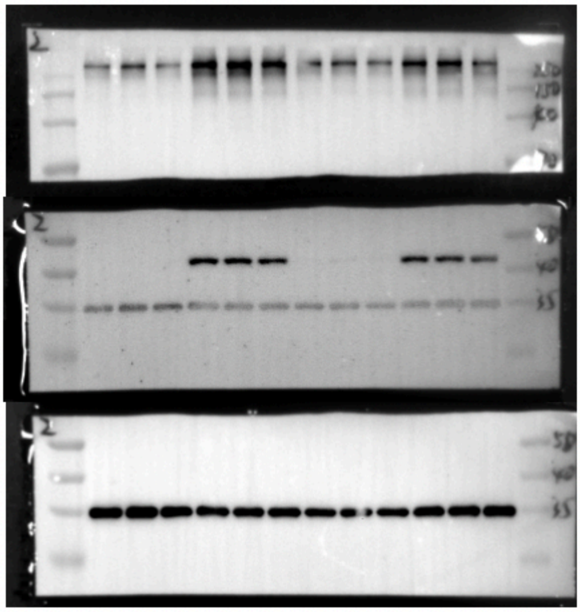

Fibronectin

asma

GAPDH

**Figure 7F**

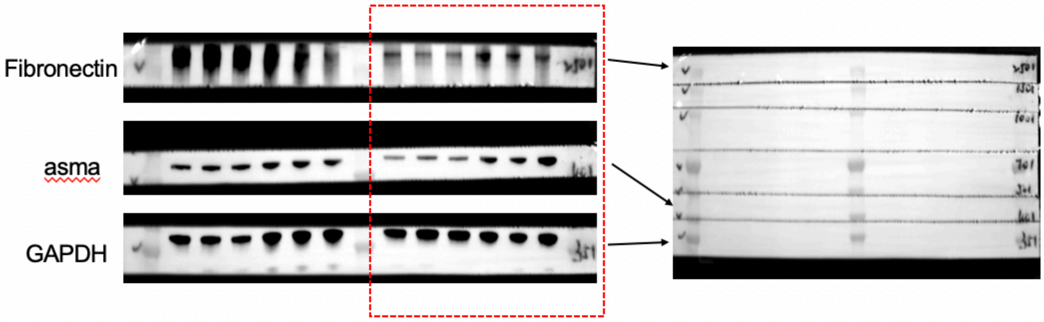


**Figure 7G**
